# Value of Functionalized Superparamagnetic Iron Oxide Nanoparticles in the Diagnosis and Treatment of Acute Temporal Lobe Epilepsy on MRI

**DOI:** 10.1155/2016/2412958

**Published:** 2016-01-27

**Authors:** Tingting Fu, Qingxia Kong, Huaqiang Sheng, Lingyun Gao

**Affiliations:** ^1^Department of Neurology, Affiliated Hospital of Jining Medical University, Guhuai Road, No. 79, Jining, Shandong 272000, China; ^2^Department of Magnetic Resonance Imaging, Affiliated Hospital of Jining Medical University, Guhuai Road, No. 79, Jining, Shandong 272000, China

## Abstract

*Purpose. *Although active targeting of drugs using a magnetic-targeted drug delivery system (MTDS) with superparamagnetic iron oxide nanoparticles (SPIONs) is a very effective treatment approach for tumors and other illnesses, successful results of drug-resistant temporal lobe epilepsy (TLE) are unprecedented. A hallmark in the neuropathology of TLE is brain inflammation, in particular the activation of interleukin-1*β* (IL-1*β*) induced by activated glial cells, which has been considered a new mechanistic target for treatment. The purpose of this study was to determine the feasibility of the functionalized SPIONs with anti-IL-1*β* monoclonal antibody (mAb) attached to render MRI diagnoses and simultaneously provide targeted therapy with the neutralization of IL-1*β* overexpressed in epileptogenic zone of an acute rat model of TLE.* Experimental Design. *The anti-IL-1*β* mAb-SPIONs were studied in vivo versus plain SPIONs and saline. Lithium-chloride pilocarpine-induced TLE models (*n* = 60) were followed by Western blot, Perl's iron staining, Nissl staining, and immunofluorescent double-label staining after MRI examination.* Results. *The magnetic anti-IL-1*β* mAb-SPION administered intravenously, which crossed the BBB and was concentrated in the astrocytes and neurons in epileptogenic tissues, rendered these tissues visible on MRI and simultaneously delivered anti-IL-1*β* mAb to the epileptogenic focus.* Conclusions. *Our study provides the first evidence that the novel approach enhanced accumulation and the therapeutic effect of anti-IL-1*β* mAb by MTDS using SPIONs.

## 1. Introduction

Temporal lobe epilepsy (TLE) is the most prevalent form of adult focal onset epilepsy (a condition characterized by recurrent, unpredictable, and spontaneous seizures) and is often associated with pharmacological resistance [1], thus medically refractory epilepsy. According to statistics, as many as 75% of patients with TLE are considered to have drug-resistant epilepsy [2], which is a condition defined by the International League Against Epilepsy as persistent seizures, in spite of having used at least two appropriate and adequate antiepileptic drug (AED) treatments [3]. Despite many other approaches, such as surgery (resection or removal of small areas of the brain where the seizures originate) [4], vagus nerve stimulation (VNS) [5, 6], electrical stimulation [7], or dietary treatment (the classic ketogenic diet and its variants) [8] to treat refractory patients, these alternative treatments all remain arguably mostly underutilized because of various reasons such as lacking early identification and referral of appropriate surgical candidates, and patients with medically refractory epilepsy are too often not referred to epilepsy centers or referred too late to prevent irreversible disability [9]. Thus, a novel effective noninvasive strategy is clearly needed. Of note, the therapeutic deficiency with respect to AEDs in patients with medically refractory epilepsy includes resistance to drugs, nonspecificity towards a pathologic site, low local concentration, nonspecific toxicity, other adverse side effects [10], and the high suicide risk [11, 12]. In the present study, we attempted to solve these shortcomings by combining anti-interleukin- (IL-) 1*β* monoclonal antibody (mAb) with the a magnetic-targeted drug delivery system (MTDS) [13–16]. In this study, anti-IL-1*β* mAb, as an anti-epileptogenic therapeutic targeting proteins, was chelated to superparamagnetic iron oxide nanoparticles (SPIONs), composed of iron oxide and polyethylene glycol (PEG), and intravenous tail injections were performed and the possibility of epileptogenic focus imaging and simultaneous targeted therapy of new drug-delivery particles using MRI providing an external magnetic field was explored in a rat model of TLE.

Previous experimental evidence supports the notion that anti-IL-1*β* mAb may be a promising antiepileptogenic therapeutic agent for TLE by acting on IL-1*β*, a novel molecular target [17, 18]. More recently, the rapidly growing body of clinical and experimental evidence has provided strong support to the hypothesis that immunity and inflammation are considered to be key elements in the pathobiology of a number of human TLE and convulsive disorders [19, 20]. A growing body of research has shown that the immune exemption of the central nervous system (CNS) is related. The CNS has a kind of special immune defense, including microglia, astrocytes, and endothelial cells, that participates in the immune response within the brain. Microglia are “the sentinel” of the CNS immune system and maintain a steady state in the CNS environment. The biological function of the microglia is similar to the peripheral system of macrophages and is inherent in immune defense within the CNS. Microglia are relatively small in number, representing approximately 5% of all glial cells. When the CNS is infected and impaired, the function of static microglia is active, becomes macrophages, and participates in the adaptive immune response with circulating T cells. Excessive activation of microglia produces neurotoxicity, which is an important source of proinflammatory factors and oxidative stress. Activated microglia cells can release toxic substances, such as cytokines. Astrocytes, one of the largest glial cells and 10-fold the number of neurons, are an important member of the nervous system, which can guide neuromobility, provide the energy for neurons, and regulate the excitability of neurons. Astrocytes are an important “energy library” in the CNS and the astrocyte network, which are connected by gap junction channels and can provide energy for neurons to maintain glutamate synaptic transmission and discharge.

Astrocytes also express some cytokine receptors, especially IL-1 receptors [21], the proinflammatory cytokine, IL-1*β*, activated intracellular signaling pathways by acting on the IL-1R, and may increase reactive glial cell proliferation and signal amplification inflammation in TLE [22, 23]. IL-1*β* can trigger the neuronal endogenous inflammatory response by activating the PI3K/Akt/mTOR signaling pathway, and activation of this pathway participates in seizure generation and pathogenesis [23]. In addition, IL-1*β* can aggravate the occurrence and development of epilepsy and can rapidly lower the focal ictal event threshold [24]. The reverse results could be obtained when blocking IL-1*β* signaling [25, 26]. These findings strengthen the possibility targeting these inflammatory pathways and IL-1*β* may represent an effective therapeutic strategy to prevent seizures.

Thus, IL-1*β* should be considered as a new molecular target in the design of AEDs, which might not only inhibit the symptoms of this disorder, but also prevent or abrogate disease pathogenesis [27]; however, the use of anti-IL-1*β* mAb as a neuroprotective therapeutic can be limited by the hindered mobility through the BBB. An increasing body of experimental evidence suggests that MTDS can overcome the BBB issue [28–30]. Guiding magnetic nanoparticles (MNPs), with the help of an external magnetic field to its target, is the principle for the development of MTDS [31, 32]. SPIONs are small synthetic *γ*-Fe_2_O_3_ (maghemite), Fe_3_O_4_ (magnetite), or *α*-Fe_2_O_3_ (hematite) particles with a core ranging from 10 to 100 nm in diameter [33]. In addition, due to the essential characteristics, SPIONs exhibit unique electronic, optical, and magnetic properties that have been widely used in* in vivo* biomedical applications [33, 34], especially MRI contrast enhancement [35, 36] and drug delivery [37], where SPIONs facilitate laboratory diagnostics and therapeutics. Further studies have demonstrated that SPIONs with proper surface architecture and conjugated targeting ligands/proteins have shown great potential in nanomedicine. For example, functionalized SPIONs conjugated to targeting ligands, such as alpha methyl tryptophan (AMT) and 2-deoxy glucose (2DG), are capable of crossing the BBB and concentrating in the epileptogenic tissues and are approved for MRI contrast agents in an epilepsy model [38, 39]. Similarly, SPIONs with drugs loaded can be guided to the desired target area (epileptogenic tissues) using an external magnetic field, while simultaneously tracking the biodistribution of the particles on MRI [40]. More specifically, the current research involving SPIONs is opening up wide horizons for their use as diagnostic agents in MRI and simultaneously as drug delivery vehicles [41].

In this study, we demonstrated the remarkable capability of anti-IL-1*β* mAb-SPION to specifically deliver neutralizing-IL-1*β* antibody into the epileptogenic zone, thus significantly increasing the efficacy of therapy and simultaneously rendering these tissues visible on MRI as a contrast-enhancing agent. The new approach, anti-IL-1*β* mAb-SPION-MRI, provides a safe theranostic platform, which integrates targeted delivery of antibody drugs and enhances MR imaging of TLE. Thus, this new approach using a functionalized SPION-MRI drugs delivery system truly makes them theranostic (therapeutic and diagnostic) [40].

## 2. Materials and Methods

### 2.1. Particles

Two types of functionalized nanoparticles (plain [P] SPIONs and anti-IL-1*β* mAb-SPIONs) were used in this study. P-SPIONs (unconjugated) were prepared by a procedure similar to that described by Akhtari et al. [38]. The particle consists of monocrystalline iron oxide cores of maghemite (*γ*-Fe_2_O_3_) coated with a PEG layer using a precipitation reaction, with an average diameter of 10–20 nm (an average iron-oxide core diameter of 2-3 nm). Anti-IL-1*β* mAb was then covalently conjugated to the plain particles with the same characteristics (solid content, 5 mg/mL; iron concentration, 2.4 mg/mL; antibody concentration, 10 *μ*g/mg Fe). All of the particles used for this study were provided by MicroMod Company (Rostock, Germany).

### 2.2. Experimental Animals

All procedures described in this study were approved by the Medical University of Jining Institutional Animal Care and Use Committee. Sprague-Dawley rats (8–10 weeks old; LuKang Pharmaceutical Co., Shandong, China), weighing 250–300 g, were used in all experiments. All rats were housed individually or in pairs at an ambient temperature of 20–25°C and relative humidity of 50%–60% and 12 h light/dark cycle with access to food and water* ad libitum. *All studies were performed in the Key Laboratory of Molecular Pathology of Jining Medical University (Shandong, China).

### 2.3. Pilocarpine-Status Epilepticus (SE) Model

127 mg/kg of lithium chloride (Sigma-Aldrich, St. Louis, MO, USA) was administered to all of the rats via a peritoneal injection. After 18–20 h, the rats were injected with a low dose of methylscopolamine (1 mg/kg intraperitoneally in 0.9% NaCl; Sigma-Aldrich) 15–30 min before pilocarpine (Sigma-Aldrich) injections to minimize peripheral effects of cholinergic stimulation. A single dose of pilocarpine was administered (330–345 mg/kg intraperitoneally in 0.9% saline) following 30 min later to induce SE. The period of SE was alleviated after 90 min with 1 diazepam injection (10 mg/kg in saline, intraperitoneal; Xupu Pharma, China). The rats were observed for 1 h and scored according to a modified Racine seizure scale [42] as follows: 1 = facial movements; 2 = head nodding and chewing; 3 = unilateral forelimb clonus; 4 = bilateral forelimb clonus; and 5 = bilateral forelimb clonus, rearing, and loss of balance. The models of TLE that scored ≥ 4 were employed in the following study. All animals were injected with 5% dextrose in lactate Ringer's solution (5 mL intraperitoneal bid) and hand-fed moistened cookies for approximately 3 days and thereafter when needed. Then, randomly select 45 of the successful and survival rat epileptic models, then divided into 3 groups (saline control group (*n* = 15), P-SPIONs control group (*n* = 15), and the anti-IL-1*β* mAb-SPION group (*n* = 15)).

### 2.4. MRI Study

MRI was performed using a clinical 3.0 T MRI scanner (Siemens Magnetom, version 3.0 T; Berlin, Germany) along with general 3-inch circular coil at room temperature. T2-weighted images were acquired using the following parameters: repetition time (TR)/echo time (TE), 2500/70; 6 echoes, 192*∗*192; slices, 12; thickness slice, 2.0 mm; field of view (FOV), 80 mm; and acquisition time, 6.5 min. MRI scans were acquired on all models 72 h after SE (acute state). MR images were acquired before the injection of anti-IL-1*β* mAb-SPION (15 mg/kg), plain SPIONs (15 mg/kg), and saline (equivalent dose) in the tail vein 72 h after SE as well as ~4 h after tail vein injection to compare the differences between images. All animals were denied food for at least 12 h before injection. T2 relaxation data were acquired using T2 mapping.

### 2.5. Tissue Processing

After the MRI study, mice (*n* = 10 per group) were anesthetized and perfused transcardially using 0.9% saline followed by 0.1% sodium sulfide and 4% paraformaldehyde (0.1 M (pH 7.4)). Brains were immediately removed after perfusion and immersed in 4% paraformaldehyde overnight for additional fixation followed by being arranged to sink in 20% and/or 30% sucrose solution in PBS for 24 h at 4°C. Brains were extracted and were cut on a freezing sliding microtome at a thickness of 5 *μ*m with consecutive coronal slices from all brains throughout the septotemporal extension of the hippocampus for neuropathology. One slice was taken for every interval of 6 and a total of 10 slices were taken from each specimen. Two slices were randomly selected, respectively, for Perl's iron staining, Nissl staining, FJB staining, and immunofluorescence, and subjected to statistical analysis. In addition, remnant animals (*n* = 5 per group) were decapitated under anesthesia. The brains were removed rapidly and hippocampal tissues were isolated and stored in liquid nitrogen until use for Western blot analysis.

### 2.6. Western Blot

The tissue samples were dissected out at 4°C and homogenized in lysis buffer and PMSF (nos. p0013 ST506; Beyotime Institute of Biotechnology, Jiangsu, China) for 30 min and centrifuged at 12,000 g for 15 min at 4°C to separate and extract proteins. Total proteins (30 *μ*g per lane; Bio-tanon, Shanghai, China) were separated using 8%–12% sodium dodecyl sulfate (SDS) polyacrylamide gels, and 10% acrylamide and transferred to polyvinylidene fluoride (PVDF) membranes, and each sample was run in duplicate. Proteins were transferred to Millipore polyvinylidene fluoride membranes by electroblotting. The membrane was blocked with 5% nonfat milk in Tween-TBS (TBST) for 2 h at room temperature and incubated at 4°C overnight with primary antibodies against IL-1*β* (1 : 500 dilution; Santa Cruz Biotechnology Inc., Santa Cruz, CA, USA) or against IL-1R1 (1 : 500 dilution; Santa Cruz Biotechnology Inc.). After rinsing, the membranes were appropriately incubated with horseradish peroxidase- (HRP-) conjugated goat anti-rabbit IgG (1 : 5000 dilution; Santa Cruz Biotechnology Inc.) for 2 h at room temperature. Protein bands were visualized by ECL Western blot detection reagents (ECL, no. P0018; Beyotime Institute of Biotechnology) and were exposed to X-ray film. All experiments were repeated at least three times. Optical density values in each sample were normalized using the corresponding amount of *β*-actin.

### 2.7. Histology

In order to observe the distribution of iron particles in the brain tissue of each sample, Perl's iron staining of the sections was performed following the procedure of Perl's iron stain kit (Solarbio, Beijing, China). The sections were incubated in a stock potassium ferrocyanide solution (10%) for 5 min. Immediately prior to use, a working potassium ferrocyanide solution was prepared (70 mL stock potassium ferrocyanide + 30 mL 10% HCl) and applied for 20 min. Sections were counterstained with Nuclear Fast Red for 5 min.

To investigate the antiepileptic effect of anti-IL-1*β* mAb-SPIONs on the neuronal damage in the hippocampus induced by SE, Nissl staining, Fluoro-Jade (F-J) B staining, and immunofluorescent double-label staining were performed.

To evaluate the degree of hippocampal sclerosis and the overall cellular death, brain sections of each specimen underwent Nissl staining with toluidine blue according to the well-known Nissl staining protocol, and samples were dried at room temperature. After 3 washes with distilled water, the slides were dipped in 0.1% cresyl violet (Sigma-Aldrich) for 30 seconds, washed again, and then dehydrated. The sections were permeabilized with xylene and mounted with neutral resin. Four randomly chosen nonoverlapping fields were selected to calculate the number of hippocampal CA3 pyramidal cells under a light microscope (Olympus, Hamburg, Germany) at 400x magnification. The mean was taken as the average number of each type of neuron in the hippocampus. F-J B is a high affinity fluorescent marker for the localization of neuronal degeneration. The staining was performed with the following procedure: the slides were first immersed in a solution containing 1% sodium hydroxide in 80% alcohol (20 mL of 5% NaOH added to 80 mL of absolute alcohol) for 5 min. This was followed by a 2-minute immersion in 70% alcohol and 2 min in distilled water. The sections were then transferred to 0.06% potassium permanganate and then a 0.0004% F-J B solution (Millipore, Massachusetts, USA). After washing in distilled water, the sections were placed on a slide warmer at 50°C until they fully dried. The tissue was then examined using an epifluorescent microscope (ZEISS, Oberkochen, Germany) with blue (450–490 nm) excitation light and a barrier filter.

To highlight the differences in the levels of activated nuclear factor-kappa B (NF-*κ*B) p65 and astrocyte hyperplasia within the CA3 region of the hippocampus, double labeling of GFAP and NF-*κ*B p65 was carried out. The brain sections were thawed and fixed in ice-cold acetone for 1 min. Nonspecific binding was blocked using 10% normal goat serum in PBS with 0.1% Triton X-100 for 2 h. The brain sections then were incubated with primary anti-NF-*κ*B p65 antibody (1 : 500 dilution; Abcam, Shanghai, China) and astrocyte-specific primary antibody (rabbit anti-GFAP antibody-Cy3, 1 : 400 dilution; Abcam) in PBS overnight at 4°C following preincubation in 10% normal goat serum to block nonspecific binding, washed with PBS and then with a secondary antibody (Alexa Fluor 488 donkey anti-rabbit IgG antibody, 1 : 500 dilution; Abcam) for 1 h at room temperature. Finally, cells were washed three times and coverslips were mounted using Immu-Mount (Thermo Scientific, Germany). All brain sections were observed under a fluorescence microscope (ZEISS).

### 2.8. Statistical Analyses

All results are expressed as the mean ± standard deviation (SD). Analysis of variance for repeated measures was performed with SPSS 19.0 statistical software. One-way analysis of variance (ANOVA) was used for comparisons between groups, followed by the LSD test for between-group comparisons. The results of MRI T2 values before and after injection in each group were compared with *t*-tests. *P* < 0.05 was considered statistically significant and *P* < 0.01 was considered highly significant.

## 3. Results

### 3.1. MRI Studies

**Figure 1:**
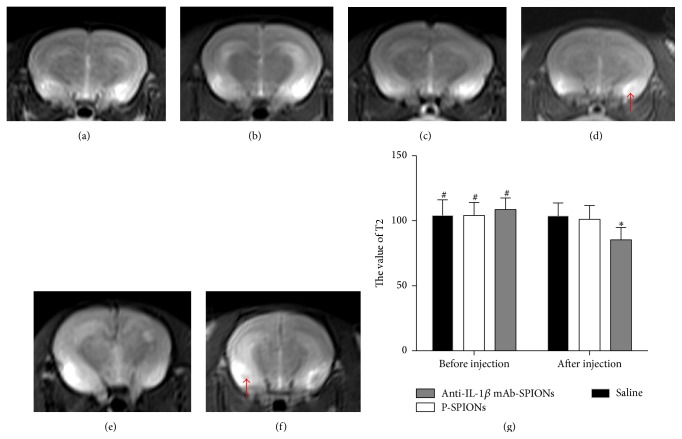
MR images of rat brains in the three groups are shown before and after tail vein injection of particles (15 mg/kg). ((a), (c), and (e)) Baseline MRI prior to injection with saline, P-SPIONs, anti-IL-1*β*, and mAb-SPIONs; (b) MR images acquired 4 h after saline injection. (d) MR image acquired 4 h after P-SPIONs injection; area of negligible negative enhancement is not visible (red arrow). (f) MR images acquired 4 h after anti-IL-1*β* mAb-SPIONs injection; area of negative enhancement is visible (red arrow) in CA3 showing unilateral uptake of particles; (g) the changes in the value of T2 before and after injection in the three groups. Data represent mean ± SD of 10 pilocarpine-SE rats per group. ^*∗*^ *P* < 0.05 versus preinjection. ^#^ *P* > 0.05 between three groups when preinjected.

Prior to particle injection, the baseline T2-weighted MR images of the brains in the three groups all showed areas of positive contrast enhancement (increased signal intensity) of acute lesions of the TLE model (Figures 1(a), 1(c), and 1(e)). We chose the side of the macroscopic lesion to carry out the statistical analysis. The MR signal intensity at the lesion site before particle injection between the three groups was not significantly different (*P* > 0.05; Figure 1(g)). The postinjection images of rats to whom anti-IL-1*β* mAb-SPION was administered (Figure 1(f)) showed areas of negative enhancement (red arrow) in the regional zone of the brain lesion, in agreement with a significant decrease in T2 values by 21.5% in comparison to the preinjection image of epilepsy (*P* < 0.01; Figure 1(g)). The hypointense region within the epileptogenic lesion is indicative of nanoparticle accumulation, which causes a reduction in signal intensity on T2-weighted image. After injection of P-SPIONs, the MR images of P-SPIONs groups showed areas of negligible negative enhancement (arrows; Figure 1(d)) of partial lesions with no significant difference in T2 values (*P* > 0.05; Figure 1(g)). The MR signal intensity of the saline group showed no significant difference in comparison to preinjection (*P* > 0.05), corresponding to almost no signal reduction within the epileptogenic tissues (Figure 1(b)).

### 3.2. Distribution of Magnetic Nanoparticles in Rat Brains of the Acute TLE Model

**Figure 2:**
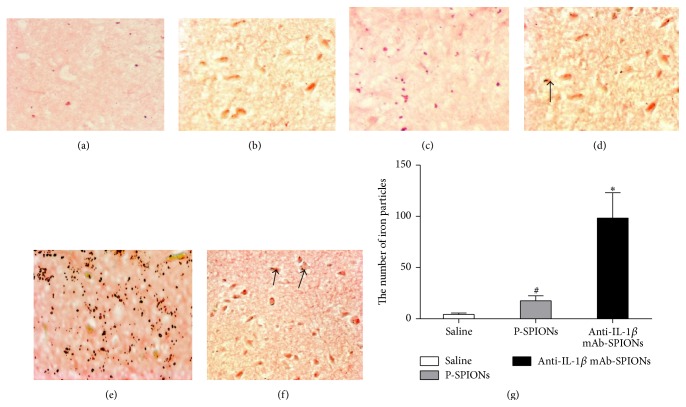
The distribution of iron particles in the hippocampus CA3 of models of TLE at 72 h post-SE after tail vein injection of particles. ((a)-(b)) Perl's iron staining of saline group; ((c)-(d)) Perl's iron staining of P-SPIONs group; ((e)-(f)) Perl's iron staining of anti-IL-1*β* mAb-SPIONs group. ((a), (c), and (e)) Magnification 40; ((b), (d), and (f)) magnification 400; (g) shows bar graphs (mean ± SD, *n* = 10) for the number of iron particles. ^*∗*^ *P* < 0.01 versus saline group. ^#^ *P* > 0.05 versus saline group and anti-IL-1*β* mAb-SPIONs group.

To confirm that the signal intensity change of epileptogenic lesions following administration of anti-IL-1*β* mAb-SPIONs was due to the accumulation of SPIONs in the epileptogenic tissues, we performed Perl's iron staining of the brain tissue after MR imaging (Figure 2). Perl's iron staining of coronal sections from brain tissues showed uptake of anti-IL-1*β* mAb-SPIONs (arrows; Figure 2(f)), crossing of the BBB, and intracellular localization in the hippocampus CA3; this accumulation manifested as areas of negative enhancement of the MRI seen in Figure 1, with statistical significance compared with the saline control group (*P* < 0.01; Figure 2(g)). Additionally, little brown iron particles were observed in the P-SPIONs control group (arrows; Figure 2(d)), and there was significant difference (*P* < 0.05) compared with the saline group.

### 3.3. Anti-IL-1*β* mAb-SPIONs: Neutralization of IL-1*β* Ameliorates Pilocarpine-Induced Epilepsy

**Figure 3:**
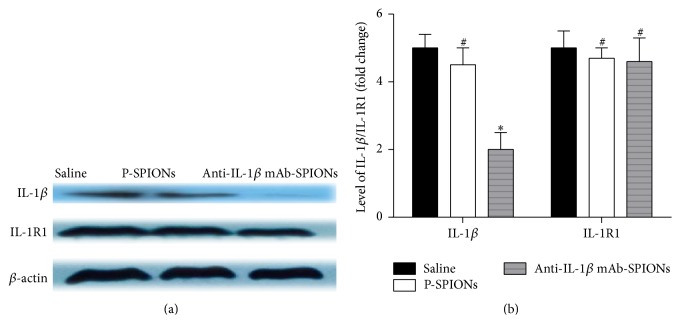
The protein expression of IL-1*β*/IL-1R1 (mean ± SD, *n* = 5) measured in the rat hippocampus after particle injection, as assessed by Western blot. (a) Western immunoblots for IL-1*β* and IL-1R1 protein expression in the saline, P-SPIONs, and anti-IL-1*β* mAb-SPIONs groups. (b) shows bar graphs for IL-1*β* and IL-1R1 protein expression plotted on the vertical-axis as a fold change relative to the saline group. ^*∗*^ *P* < 0.05 versus saline group and P-SPIONs group; ^#^ *P* > 0.05 versus saline group.

Western blot for investigating the neutralization of IL-1*β* protein and expression of IL-1R1 protein was performed (Figure 3). After injection of particles, the P-SPIONs group showed few changes (*P* > 0.05; Figure 3(b)) in the level of IL-1*β* protein and IL-1R1 protein in comparison to the saline group (Figure 3(a)); however, compared with the two control groups, the anti-IL-1*β* mAb-SPIONs-treated group had a significant decrease (*P* < 0.01) in the level of IL-1*β* protein, with no significant decrease (*P* > 0.05) in the level of IL-1R1 protein.

**Figure 4:**
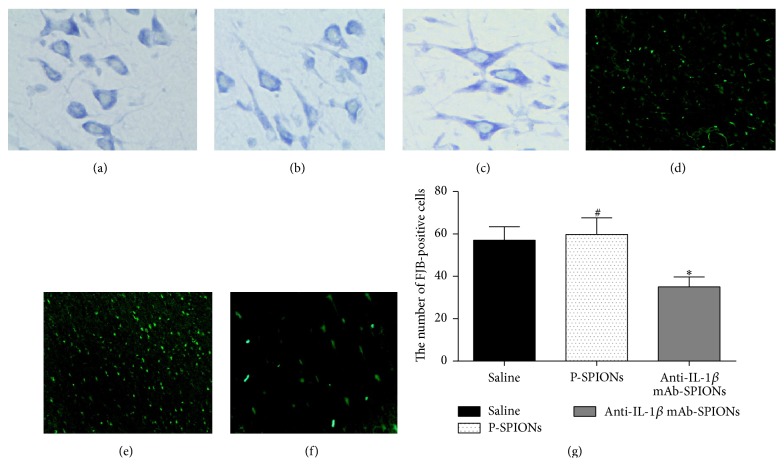
Effect of anti-IL-1*β* mAb-SPIONs dependency on SE-induced neuronal cell loss of hippocampus CA3 at 72 h post-SE after injection. ((a)–(c)) The Nissl staining (400x); ((d)-(e)) the FJB staining (40x) showed FJB-positive cells; ((a), (d)) saline group; ((b), (e)) P-SPIONs group; ((c), (f)) anti-IL-1*β* mAb-SPIONs group. (g) Data (mean ± SD, *n* = 10) present the number of the FJB-positive cells. ^*∗*^ *P* < 0.05 versus saline group and P-SPIONs group; ^#^ *P* > 0.05 versus saline group.

**Figure 5:**
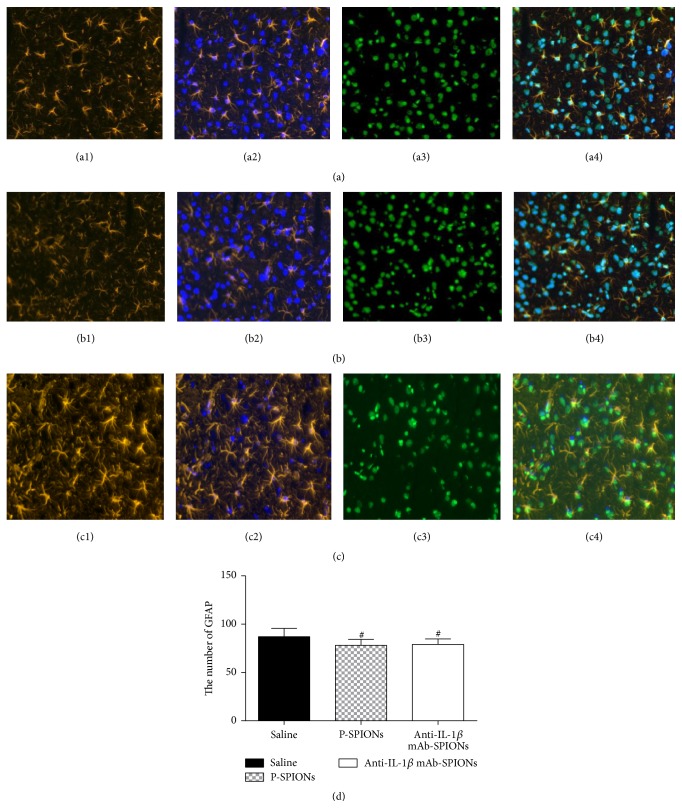
NF-kappa B p65 expression and astrocyte hyperplasia in the hippocampus CA3 of epileptic rats treated with anti-IL-1*β* mAb-SPIONs versus saline and P-SPIONs (20x); ((a1)–(b4)) show the activation of GFAP-positive astrocytes and the expression of NF-kappa B p65 in astrocyte nucleus was observed in the saline ((a1)–(a4)) and P-SPIONs groups ((b1)–(b4)). Colocalization panels ((a4)–(c4)) show NF-*κ*B p65 expression in activated astrocytes in the hippocampus CA3 of epileptic rats; (c4) notes the inhibition of NF-*κ*B p65 nucleus transfer in anti-IL-1*β* mAb-SPIONS-treated rats; (c1) shows that the astrocytes still have an activated phenotype. (d) shows the number of positive-GFAP astrocytes (mean ± SD, *n* = 10). ^#^ *P* > 0.05 versus saline group.

To explore the effect of anti-IL-1*β* mAb-SPIONs on neuronal damage of brain regions induced by pilocarpine-induced SE, Nissl staining of the brain sections was performed. Representative photomicrographs of Nissl staining results are shown in Figure 3. Nissl staining of samples revealed typical neuropathologic changes, including neuronal loss, organizational structure disorders (nucleus shrinkage or disappearance of Nissl bodies) of the hippocampal CA3 area in the control groups (saline and P-SPIONs group; Figures 4(a) and 4(b)); however, after injection of the novel drug, anti-IL-1*β* mAb-SPIONs, improvement of organizational structure in the hippocampal CA3 area was observed in the experimental group (Figure 4(c)). F-J B staining selectively marked the damaged neurons in agreement with the results of Nissl staining of the hippocampal CA3 area showing a trend towards neuronal loss. Compared to the control groups, a decrease in the number of F-J B-positive neurons was observed in the anti-IL-1*β* mAb-SPIONs group after the injection of the novel drug (*P* < 0.05; Figures 4(d)–4(g)). This extensive cell loss was accompanied by microglial proliferation and the activation of NF-*κ*B p65 in the brain hippocampus. We studied NF-*κ*B p65 expression and astrocytes hyperplasia in the hippocampus CA3 of epileptic rats treated with anti-IL-1*β* mAb-SPIONs versus saline and P-SPIONs (Figure 5). The activation of GFAP-positive astrocytes and the nuclear transfer of NF-*κ*B p65 in astrocytes were observed in saline group (Figures 5(a1)–5(a4)) and P-SPIONs group (Figures 5(b1)–5(b4)); there was no statistical significance (*P* > 0.05; Figure 5(d)) between groups. Compared with the control groups, the model rats of TLE treated with anti-IL-1*β* mAb-SPIONs showed the inhibition of  NF-*κ*B p65 expressed in the nuclei of astrocytes of hippocampus CA3 (Figure 5(c4)). Although the cell nucleus of astrocytes did not express NF-*κ*B p65 during antibody treatment, the astrocytes still showed an activated phenotype (Figures 5(c1) and 5(d)).

## 4. Discussion 

The results of this study provide important proof of principle for several aspects of the hypothesis that anti-IL-1*β* mAb-SPIONs, which cross the BBB, can be used to localize and delineate specific cerebral functions, such as the epileptogenic focus, and simultaneously have a powerful targeted anti-epileptic effect after tail vein administration in a rat model of acute seizures with neuropathologic features mimicking TLE. Most importantly, the improvement of neuropathology and neurotomy (decreased neuronal cell loss and astrocyte proliferation and inhibition of the NF-*κ*B p65 activation) of epileptogenic tissues in rat brain provides evidence that the novel treatment showed high penetration of the antibody into the epileptogenic zone of the brain hippocampus of epileptic rats.

In the present study, we first focused on the uptake of the SPIONs particles and then the ability of crossing the BBB in the brain by the method of tracking even small amounts of magnetite-labeled nanoparticles in the rat brain using MRI [43] and Perl's iron staining. The T2-negative enhancement in epileptogenic tissues of the MRI patterns performed before and after anti-IL-1*β* mAb-SPIONs injection confirms previous results showing that these functionalized magnetized particles were taken up by brain parenchyma and then crossed the BBB in this epilepsy model [38, 44]. The results of the distribution of particles in the brain by Perl's iron staining showed that the number of iron particles of epileptogenic tissues in the experimental group exceeds the control groups, corresponding to the negative enhancement changes of MRI images. As a macromolecule, anti-IL-1*β* mAb cannot aggregate sufficiently to the brain because of an inability to cross the BBB. The results of the present* in vivo* study indicated that MTDS significantly improved the accumulation of the anti-IL-1*β* mAb in pathologic sites and decreased the undesirable side effects. The mechanisms underlying anti-IL-1*β* mAb-SPIONs crossing the BBB accounted for a number of observations. First, the inflammation induced by epileptic seizures and epileptic activity itself can result in BBB dysfunction (the disturbances of its integrity and functionality), including an increase in cerebral capillary permeability [45] and an increase in pinocytosis at the level of the cerebral endothelium [46]. Second, the inherent property of nanoparticles, such as the small size, can stick to the cell surface and participate in the material transfer between cells [30]. Previous evidence has shown that antibody-mediated targeting of iron oxide nanoparticles can cross the BBB by the method of receptor-mediated transcytosis [47]. Therefore, monoclonal antibodies, when chelated to the SPIONs, not only influence the specificity of drug delivery, but also penetrate and distribute anti-IL-1*β* mAb-SPIONs by rendering them capable of selectively binding certain antigens (IL-1*β*) overexpressed on epileptogenic tissues. The external magnetic field of MTDS, with the major advantage of combining simplicity, a modest cost, enhanced localization of deficient delivery, and reductions in both incubation time and vector doses [31], has directed the delivery of drug coupled to magnetic particles to further enhance selective brain deposition [32]. Recent information suggests that using a combined strategy of ultrasound, magnetic targeting, and drug-loaded nanoparticles can also improve the outcome of targeted delivery of chemotherapy drugs to the brain [48, 49]. The fact that SPIONs should be considered as a strong T2 contrast-enhancing agent, certified by the phantom study, is suggested by changes in negative enhancement between the MRI pattern seen with anti-IL-1*β* mAb-SPIONs and plain SPIONs or saline during the acute phase.

Although a number of previous studies have shown that IL-1*β* contributes to epileptic seizures and can be considered as a therapeutic target for TLE, few of the studies have attempted to use specific neutralizing antibody in the treatment of TLE. Here, we found the potential neuroprotective and anti-epileptic effect of anti-IL-1*β* mAb. In this study, the downregulation of IL-1*β* protein in Western blot after novel drug injection indicated that anti-IL-1*β* mAb, which binds tightly to IL-1*β* with a neutralization potency > 10 times higher than the marketed antibody, canakinumab [50], was highly sensitive and specific for IL-1*β* protein overexpressed in epileptogenic tissues. Previous evidence showed that neuroinflammation, triggered by the activation of the IL-1*β*/IL-1 receptor type 1 (IL-1R1) axis, played a key role in epileptogenic brain areas. IL-1R1-mediated posttranscriptional signaling in neurons, which was induced by IL-1*β*, promoted hyperexcitability, seizures, and excitotoxicity by enhancing neuronal Ca^2+^ influx. However, this study showed no statistical significance in expression of IL-1R1 protein after antibody treatment. Even so, this study demonstrated that neutralization of IL-1*β* simultaneously led to neuroprotective effects, remarkable reduction of hippocampal neuronal loss, and the improvement of neuronal organizational structure, on SE-associated neuronal damage in the CA3 area of the hippocampus as well as performing targeted location on MRI. The combined sensitivity and specificity of anti-IL-1*β* mAb-SPIONs provided a robust and safe theranostic platform for TLE.

Among the classical AEDs and the nonclassical antiseizure drugs, vinpocetine and carbamazepine, with a mechanism of action that involves a decrease in Na^+^ channel permeability, were investigated and shown to be highly effective in reducing the cerebral inflammatory IL-1*β* expression to render reducing the increased brain excitability accompanying seizures [51]. The neutralizing anti-interleukin-1*β* antibodies, as an anti-inflammatory drug, also have significant positive effects on other pathologic conditions. Recently, some researchers have found that in the fetus, the anti-IL-1*β* mAb which be taken up by brain in ischemia-reperfusion injury, can then attenuates ischemia-reperfusion related fetal BBB dysfunction [52]. In traumatic brain injury (TBI), similar to our studies, the neutralization of IL-1*β* was associated with improved histologic and cognitive outcome [53], modification of the inflammatory response, reduced loss of hemispheric tissue, and attenuation of the microglial activation caused by TBI [54]. However, in the present study, the behavioral analysis of epileptic rats after antibody treatment was not performed.

To evaluate potential mechanisms of action for the anti-inflammatory antibody, we then used immunofluorescent double-labeling staining of the CA3 region of the hippocampus to evaluate changes in activated NF-*κ*B p65 and astrocyte hyperplasia induced by SE. The experimental results showed that the role of anti-IL-1*β* mAb-SPIONs on treatment for epilepsy is not affected by decreased proliferation of astrocytes, implying that astrocytes which affect the development of epilepsy are related to many mechanisms, such as ion channels and water channels, amino acid metabolism of the excitatory, and inhibitory amino acids in the inside and outside of cells, cell factors, and gap junctions. In terms of ion channels and water channels, previous study showed that astrocytes lost the ability of removal of K in patients with temporal lobe epilepsy and the marked increase in the neurons in a highly excited state [55]. In addition, the experiments have proved the existence of glutamate receptors on AST cell membranes, which is a type of excitatory neurotransmitter associated with seizures, and extracellular glutamate levels in the brain are increased. Glutamic acid can stimulate a paroxysmal depolarization offset [56], leading to an epileptic discharge. A study confirmed that IL-1 may reduce the production of glutamine by reducing Glu transporter activity, indirectly reducing Glu synthesis. IL-1*β* and high mobility group protein 1 (high mobility group box 1 protein (HMGB1)) may play a role in promoting the epileptic attack by making N-methyl-D-aspartate 2 B (N-methyl-D-aspartate (NMDA2B)) phosphorylation [57].

It is generally known that anti-inflammatory response and antiepileptic effect are complementary to each other. These experimental results suggest that the antiepileptic effect of anti-IL-1*β* mAb-SPIONs, which have decreased the number of IL-1*β* molecules, was realized by reducing the reaction of IL-1*β* with receptors on the surface of the astrocytes and then reduced the activation of inflammation in the cell signaling pathways in order for the inhibition of the occurrence of epilepsy by reducing the signal of the inflammation. Previous study showed that the downstream mediators of IL-1*β* signaling, for example, the activation of NF-*κ*B, are attenuated and inhibition of nucleus transfer after neutralizing IL-1*β* after injection of antibody is associated with IL-1*β* upregulation following SE. These data are similar to previous evidence [58] and suggested that the proinflammatory cytokine, IL-1*β*, contributes to the process leading to increased cell death following TLE and is a key regulator of acute inflammatory processes in the CNS. Despite inflammatory processes, IL-1*β* was involved in the entire process of epilepsy; however, the postictal suppression (PS) phase, a common and important period following SE, is an important period of IL-1*β* action. Thus, only the acute stage (24 h after SE) was discussed. Nevertheless, the optimum concentration of anti-IL-1*β* mAb-SPIONs warrants further study.

## 5. Conclusion

In summary, this study provides the first direct evidence of the feasibility of a novel approach, integration of the anti-inflammatory drug, and functionalized SPIONs (MTDS), which crosses the BBB, in the simultaneous diagnosis and therapy of epileptic rats in acute stage of TLE* in vivo*. Magnetic field-guided delivery of the anti-IL-1*β* mAb-SPIONs enabled much more efficient uptake of SPIONs by brain tissues, significantly enhancing the neuroprotective effect of the delivered anti-inflammatory drug neutralizing-IL-1*β* antibody. Moreover, the anti-IL-1*β* mAb-SPIONS displayed much higher MRI T2 sensitivity than plain SPIONs, which makes them a very advantaged and safe therapeutic-diagnostic platform for simultaneous magnetic-targeted drugs and MRI diagnosis of other CNS diseases. Further* in vivo* studies to explore this potential are currently under way in our laboratory.

## References

[1] Kwan P., Arzimanoglou A., Berg A. T. (2010). Definition of drug resistant epilepsy: consensus proposal by the ad hoc Task Force of the ILAE Commission on Therapeutic Strategies. *Epilepsia*.

[2] Espinosa-Jovel C. A., Sobrino-Mejía F. E. (2015). Drug resistant epilepsy. Clinical and neurobiological concepts. *Revista de Neurologia*.

[3] Espinosa-Jovel C. A., Sobrino-Mejia F. E. (2015). Drug resistant epilepsy. Clinical and neurobiological concepts. *Revue Neurologique*.

[4] Gomez-Alonso J., Bellas-Lamas P. (2015). Surgical treatment for drug-resistant epilepsy. *The Journal of the American Medical Association*.

[5] Nemeroff C. B., Mayberg H. S., Krahl S. E. (2006). VNS therapy in treatment-resistant depression: clinical evidence and putative neurobiological mechanisms. *Neuropsychopharmacology*.

[6] Herring M. (2006). Commentary on ‘vagus nerve stimulation therapy for treatment of drug-resistant epilepsy and depression’. *Perspectives in Vascular Surgery and Endovascular Therapy*.

[7] Chambers A., Bowen J. M. (2013). Electrical stimulation for drug-resistant epilepsy—an evidence-based analysis. *Ontario Health Technology Assessment Series*.

[8] Yuen A. W. C., Sander J. W. (2014). Rationale for using intermittent calorie restriction as a dietary treatment for drug resistant epilepsy. *Epilepsy and Behavior*.

[9] Engel J. J. (2014). Approaches to refractory epilepsy. *Annals of Indian Academy of Neurology*.

[10] Greenwood R. S. (2000). Adverse effects of antiepileptic drugs. *Epilepsia*.

[11] Bates K. (2015). Epilepsy: current evidence-based paradigms for diagnosis and treatment. *Primary Care: Clinics in Office Practice*.

[12] Bagary M. (2011). Epilepsy, antiepileptic drugs and suicidality. *Current Opinion in Neurology*.

[13] Kubo T., Sugita T., Shimose S., Nitta Y., Ikuta Y., Murakami T. (2000). Targeted delivery of anticancer drugs with intravenously administered magnetic liposomes in osteosarcoma-bearing hamsters. *International Journal of Oncology*.

[14] Kato T., Nemoto R., Mori H. (1984). Magnetic microcapsules for targeted delivery of anticancer drugs. *Applied Biochemistry and Biotechnology*.

[15] Mardinoglu A., Cregg P. J. (2015). Modelling the effect of SPION size in a stent assisted magnetic drug targeting system with interparticle interactions. *The Scientific World Journal*.

[16] Zhang J., Misra R. D. K. (2007). Magnetic drug-targeting carrier encapsulated with thermosensitive smart polymer: core-shell nanoparticle carrier and drug release response. *Acta Biomaterialia*.

[17] Kaminski R. M., Rogawski M. A., Klitgaard H. (2014). The potential of antiseizure drugs and agents that act on novel molecular targets as antiepileptogenic treatments. *Neurotherapeutics*.

[18] Falip M., Salas-Puig X., Cara C. (2013). Causes of CNS inflammation and potential targets for anticonvulsants. *CNS Drugs*.

[19] Galic M. A., Riazi K., Pittman Q. J. (2012). Cytokines and brain excitability. *Frontiers in Neuroendocrinology*.

[20] Vezzani A. (2008). Innate immunity and inflammation in temporal lobe epilepsy: new emphasis on the role of complement activation. *Epilepsy Currents*.

[21] Rizzi M., Perego C., Aliprandi M. (2003). Glia activation and cytokine increase in rat hippocampus by kainic acid-induced status epilepticus during postnatal development. *Neurobiology of Disease*.

[22] Dube C., Vezzani A., Behrens M., Bartfai T., Baram T. (2005). Interleukin-1beta contributes to the generation of experimental febrile seizures. *Annals of Neurology*.

[23] Xiao Z., Peng J., Yang L., Kong H., Yin F. (2015). Interleukin-1*β* plays a role in the pathogenesis of mesial temporal lobe epilepsy through the PI3K/Akt/mTOR signaling pathway in hippocampal neurons. *Journal of Neuroimmunology*.

[24] Chiavegato A., Zurolo E., Losi G., Aronica E., Carmignoto G. (2014). The inflammatory molecules IL-1*β* and HMGB1 can rapidly enhance focal seizure generation in a brain slice model of temporal lobe epilepsy. *Frontiers in Cellular Neuroscience*.

[25] Ravizza T., Noé F., Zardoni D., Vaghi V., Sifringer M., Vezzani A. (2008). Interleukin converting enzyme inhibition impairs kindling epileptogenesis in rats by blocking astrocytic IL-1*β* production. *Neurobiology of Disease*.

[26] Vezzani A., Viviani B. (2015). Neuromodulatory properties of inflammatory cytokines and their impact on neuronal excitability. *Neuropharmacology*.

[27] Vezzani A., French J., Bartfai T., Baram T. Z. (2011). The role of inflammation in epilepsy. *Nature Reviews Neurology*.

[28] Yim Y. S., Choi J., Kim G. T. (2012). A facile approach for the delivery of inorganic nanoparticles into the brain by passing through the Blood–Brain Barrier (BBB). *Chemical Communications*.

[29] Tajes M., Ramos-Fernández E., Weng-Jiang X. (2014). The blood-brain barrier: structure, function and therapeutic approaches to cross it. *Molecular Membrane Biology*.

[30] Vidu R., Rahman M., Mahmoudi M., Enachescu M., Poteca T. D., Opris I. (2014). Nanostructures: a platform for brain repair and augmentation. *Frontiers in Systems Neuroscience*.

[31] Estelrich J., Escribano E., Queralt J., Busquets M. (2015). Iron oxide nanoparticles for magnetically-guided and magnetically-responsive drug delivery. *International Journal of Molecular Sciences*.

[32] Wiedmann T., Yuanyuan X., Pengyun Z. (2009). Magnetic targeted drug delivery. *Songklanakarin Journal of Science and Technology*.

[33] Mahmoudi M., Sant S., Wang B., Laurent S., Sen T. (2011). Superparamagnetic iron oxide nanoparticles (SPIONs): development, surface modification and applications in chemotherapy. *Advanced Drug Delivery Reviews*.

[34] Yoffe S., Leshuk T., Everett P., Gu F. (2013). Superparamagnetic iron oxide nanoparticles (SPIONs): synthesis and surface modification techniques for use with MRI and other biomedical applications. *Current Pharmaceutical Design*.

[35] Smith B. R., Heverhagen J., Knopp M. (2007). Localization to atherosclerotic plaque and biodistribution of biochemically derivatized superparamagnetic iron oxide nanoparticles (SPIONs) contrast particles for magnetic resonance imaging (MRI). *Biomedical Microdevices*.

[36] Mahajan S., Koul V., Choudhary V., Shishodia G., Bharti A. C. (2013). Preparation and in vitro evaluation of folate-receptor-targeted SPION-polymer micelle hybrids for MRI contrast enhancement in cancer imaging. *Nanotechnology*.

[37] Wahajuddin, Arora S. (2012). Superparamagnetic iron oxide nanoparticles: magnetic nanoplatforms as drug carriers. *International Journal of Nanomedicine*.

[38] Akhtari M., Bragin A., Cohen M. (2008). Functionalized magnetonanoparticles for MRI diagnosis and localization in epilepsy. *Epilepsia*.

[39] Akhtari M., Bragin A., Moats R., Frew A., Mandelkern M. (2012). Imaging brain neuronal activity using functionalized magnetonanoparticles and MRI. *Brain Topography*.

[40] Shubayev V. I., Pisanic T. R., Jin S. (2009). Magnetic nanoparticles for theragnostics. *Advanced Drug Delivery Reviews*.

[41] Wang C., Ravi S., Garapati U. S. (2013). Multifunctional Chitosan Magnetic-Graphene (CMG) nanoparticles: a theranostic platform for tumor-targeted co-delivery of drugs, genes and MRI contrast agents. *Journal of Materials Chemistry B*.

[42] Racine R. J. (1972). Modification of seizure activity by electrical stimulation: I. After-discharge threshold. *Electroencephalography and Clinical Neurophysiology*.

[43] Martínez Vera N. P., Schmidt R., Langer K. (2014). Tracking of magnetite labeled nanoparticles in the rat brain using MRI. *PLoS ONE*.

[44] Loureiro J. A., Gomes B., Coelho M. A. N., Do Carmo Pereira M., Rocha S. (2014). Targeting nanoparticles across the blood-brain barrier with monoclonal antibodies. *Nanomedicine*.

[45] Sheen S. H., Kim J.-E., Ryu H. J., Yang Y., Choi K.-C., Kang T.-C. (2011). Decrease in dystrophin expression prior to disruption of brain-blood barrier within the rat piriform cortex following status epilepticus. *Brain Research*.

[46] Oby E., Janigro D. (2006). The blood-brain barrier and epilepsy. *Epilepsia*.

[47] Griswold K., Ndong C., Toraya-Brown S. (2015). Antibody-mediated targeting of iron oxide nanoparticles to the folate receptor alpha increases tumor cell association in vitro and in vivo. *International Journal of Nanomedicine*.

[48] Deng C. X., Huang X. (2011). Improved outcome of targeted delivery of chemotherapy drugs to the brain using a combined strategy of ultrasound, magnetic targeting and drug-loaded nanoparticles. *Therapeutic Delivery*.

[49] Chen C. C., Sheeran P. S., Wu S.-Y., Olumolade O. O., Dayton P. A., Konofagou E. E. (2013). Targeted drug delivery with focused ultrasound-induced blood-brain barrier opening using acoustically-activated nanodroplets. *Journal of Controlled Release*.

[50] Goh A. X. H., Bertin-Maghit S., Yeo S. P. (2014). A novel human anti-interleukin-1*β* neutralizing monoclonal antibody showing in vivo efficacy. *mAbs*.

[51] Gómez C. D., Buijs R. M., Sitges M. (2014). The anti-seizure drugs vinpocetine and carbamazepine, but not valproic acid, reduce inflammatory IL-1*β* and TNF-*α* expression in rat hippocampus. *Journal of Neurochemistry*.

[52] Chen X., Sadowska G. B., Zhang J. (2015). Neutralizing anti-interleukin-1*β* antibodies modulate fetal blood-brain barrier function after ischemia. *Neurobiology of Disease*.

[53] Clausen F., Hånell A., Björk M. (2009). Neutralization of interleukin-1*β* modifies the inflammatory response and improves histological and cognitive outcome following traumatic brain injury in mice. *European Journal of Neuroscience*.

[54] Clausen F., Hånell A., Israelsson C. (2011). Neutralization of interleukin-1*β* reduces cerebral edema and tissue loss and improves late cognitive outcome following traumatic brain injury in mice. *European Journal of Neuroscience*.

[55] Das A., Wallace G. C., Holmes C. (2012). Hippocampal tissue of patients with refractory temporal lobe epilepsy is associated with astrocyte activation, inflammation, and altered expression of channels and receptors. *Neuroscience*.

[56] Siva N. (2005). Astrocytes have a key role in epilepsy. *The Lancet Neurology*.

[57] Karki P., Smith K., Johnson J., Aschner M., Lee E. Y. (2015). Genetic dys-regulation of astrocytic glutamate transporter EAAT2 and its implications in neurological disorders and manganese toxicity. *Neurochemical Research*.

[58] Diamond M. L., Ritter A. C., Failla M. D. (2014). IL-1*β* associations with posttraumatic epilepsy development: a genetics and biomarker cohort study. *Epilepsia*.

